# MLR Corresponds to the Functional Status of Monocytes in Chronic Lymphocytic Leukemia

**DOI:** 10.1155/ijin/4443773

**Published:** 2025-08-08

**Authors:** Wioleta Grzegorzewska, Michał Zarobkiewicz, Katarzyna Jastrzębska-Pawłowska, Natalia Lehman, Waldemar Tomczak, Magdalena Mizerska-Kowalska, Agnieszka Bojarska-Junak, Jacek Roliński

**Affiliations:** ^1^Department of Clinical Immunology, Medical University of Lublin, Lublin 20-093, Poland; ^2^Department of Neurosurgery and Paediatric Neurosurgery, Medical University of Lublin, Lublin 20-954, Poland; ^3^Department of Haematooncology and Bone Marrow Transplantation, Medical University of Lublin, Lublin 20-080, Poland; ^4^Department of Virology and Immunology, Institute of Biological Sciences, Maria Curie-Skłodowska University, Akademicka 19 Street, Lublin 20-033, Poland

## Abstract

**Background:** The role of the inflammatory microenvironment in initiating and progressing chronic lymphocytic leukemia (CLL) is still not clarified. To date, it has been shown that the only way to reflect inflammation in the systemic circulation is to assess inflammatory markers in peripheral blood. However, in the age of modern technology, a more detailed analysis of inflammatory cells circulating in the blood of CLL patients would be useful.

**Objectives:** The study aimed to evaluate the relationship between one of the hematological inflammatory indexes—the monocyte/lymphocyte ratio (MLR) and the risk of CLL progression associated with disease activity. In addition, we wanted to analyze whether the MLR parameter in CLL could suggest the functional immune status of circulating main monocyte subsets.

**Methods:** The study included peripheral blood samples from 54 untreated, newly diagnosed CLL patients and 20 healthy volunteers (HVs). Immunological characterization of monocyte subpopulations included their detailed assessment by multiparametric flow cytometry, including evaluation of surface markers and intracellular expression of cytokines. In addition, the relative expression of selected microRNA (miR-21-3p, miR-150-5p, miR-106a-5p) was determined in FACS-sorted monocyte subsets.

**Results:** In our study, CLL patients had significantly lower values of MLR parameters compared to HVs (*p* < 0.0001). However, the value of MLR was higher in CLL patients with negative clinical and laboratory prognostic factors, i.e., increased percentage of CD5+/CD19+ cells with ZAP-70 and CD38 expression. We noticed that the percentage of intermediate monocytes is significantly higher, but classical and nonclassical ones are significantly lower in MLR-high compared to MLR-low CLL patients. Moreover, among the monocyte subsets circulating in the blood of MLR-high, ZAP-70+, and CD38+, CLL patients' intermediate monocytes were characterised by increased intracellular expression of IL-10 and decreased miR-150-5p relative expression compared to intermediate monocytes in the MLR-low, ZAP-70-, and CD38- groups, suggesting a potential link between hematological inflammatory index and the formation of intermediate monocytes that promote CLL burden.

**Conclusions:** The MLR index may serve not only as a marker of CLL activity, but also indirectly indicate changes in the phenotype and function of monocyte subpopulations present in the blood microenvironment. Moreover, the MLR-high parameter seems to correspond to an increase in the percentage of intermediate monocytes with anti-inflammatory properties, which may potentially promote disease progression and worsen its prognosis.

## 1. Introduction

The influence of inflammation and tumor microenvironment (TME) on the pathogenesis and progression of chronic lymphocytic leukemia (CLL) remains poorly understood [1–3]. CLL is often discovered incidentally during a routine complete blood count (CBC) in adults. An elevated absolute lymphocyte count (ALC) is observed in CLL patients and is often used as a surrogate test for B-cell counts in CLL patients [4, 5]. A CBC is performed at regular intervals in all CLL patients so ALC, absolute neutrophil count (ANC), and absolute monocyte count (AMC) are obtained systematically [4]. This has led researchers to develop indices that indirectly quantify inflammation by measuring, for example, the absolute neutrophil-to-lymphocyte ratio (NLR) and absolute monocyte-to-lymphocyte ratio (MLR), as well as systemic assessment of changes in peripheral blood leukocytes [6]. The last data suggests ambiguous interactions between chronic inflammatory microenvironment cells and CLL cells to favor leukemic cell survival [7, 8]. One hypothesis suggests that with a continuous accumulation of leukemic B cells, the number of noncancerous, inflammatory cells, such as T cells and NK cells, is reduced [9, 10]. Interestingly, this observation is not obvious in the context of the monocytes. Monocytes are a population of white blood cells that remain of marginal importance in the eyes of clinicians. Despite their small numbers, monocyte contribution to the mechanisms of the innate and acquired immune response is underrated [11]. Monocytes are usually divided into three subsets: 85%–90% belong to classical (CD14++CD16−SLAN−), ∼10% to nonclassical monocytes (CD14+CD16++SLAN+), and ∼5% to intermediate monocytes (CD14++CD16+SLAN−) [12–14]. They are involved in the inflammatory response, have the capacity for phagocytosis and antigen presentation, and produce many cytokines [14]. In contrast to other inflammatory cells, the higher absolute number of monocytes in blood CLL patients is associated with a worse prognosis and shorter survival [15]. The mechanisms which directly explain the association between high AMC and the more aggressive form of CLL are not entirely clear. This quantitative difference between monocytes and other inflammatory cells in CLL may suggest an ability of monocyte subsets to change their immunological nature in response to CLL signals. The reasons for the phenotypic and cytokine-dependent pro- versus anti-inflammatory heterogeneity of monocytes in CLL remain unclear. An increasing number of studies suggest that epigenetic mechanisms play an important role in regulating the monocyte phenotype [16, 17]. One of the main epigenetics modulators are the short noncoding RNAs—microRNAs (miRNAs, miRs) [18]. miRNAs play an important role in controlling both the activation and differentiation of monocytes into macrophages and their role during inflammation [19, 20]. Intracellular microRNAs are increasingly being described in hematological malignancies, including CLL in the context of the biological function of leukemic cells, but the role of miRNAs in the function of nonleukemic cells, that is monocyte subpopulations, is still unknown [21–23]. This study aimed to evaluate the inflammatory hematological score—MLR in CLL patients with varying risks of progression. In addition, we wanted to check whether the value of MLR in CLL patients represents only the number of monocytes or can also suggest their functional immune status. Our ultimate goal was to investigate changes in the proportion of classical, intermediate, and nonclassical monocytes with intracellular expression of pro- and anti-inflammatory cytokine (TNF and IL-10) and selected miRNA (miR-106a-5p, miR-150-5p, and miR-21-3p) in CLL patients with a high and low value of MLR.

## 2. Materials and Methods

The study protocol was approved by the Bioethics Committee of the Medical University of Lublin (decision no. KE-0254/14/2018, KE-0254/113/2021). Each eligible person in the study and control groups was informed of the purpose of the research and gave informed written consent to participate and to have their clinical and laboratory data analyzed in the research project.

### 2.1. Characteristics of the Study Participants

The study group included 54 CLL patients. The study population was recruited between February 2018 and December 2023 at the Department of Hematooncology and Bone Marrow Transplantation at the Medical University of Lublin according to the latest standard criteria developed by the International Workshop on Chronic Lymphocytic Leukemia (iwCLL) [5]. Patients had not previously received chemotherapy. The study group included 35 men (65%) and 19 women (35%). The age of the CLL patients ranged from 43 to 88 years (median: 67 years).

The inclusion criteria of the study are age: adults, diagnosed with CLL based on the iwCLL, [5], without signs of infection. The exclusion criteria are diagnosed with cancer other than CLL, taking antibiotics or other medicines that affect the immune system within 3 months of the test, being diagnosed with autoimmune, allergic disease, pregnancy, blood transfusion in a month before the study, and failure to consent to participation in the study.

The stage of clinical progression of the disease was determined according to the classification of Rai et al. [24] Stage 0 was diagnosed in 19 patients, Stage I in 11, Stage II in 14, Stage III in 5, and Stage IV in 5 patients. Patients were divided into three risk groups according to the clinical progression of CLL:

• Low-risk group (Rai Stage 0)—19 patients
• Intermediate risk group (Rai Stages I–II)—25 patients
• High-risk group (Rai Stages III–IV)—10 patients

The follow-up time of patients with CLL ranged from 0.5 to 60 months.

The control group consisted of 20 healthy volunteers (10 men and 10 women) whose ages ranged from 35 to 82 years (median: 58 years). Inclusion criteria for the control group were: no oncological history, no autoimmune disease, no psychiatric disease, no pregnancy, and no transfusion in the 3 months prior to the study. In addition, healthy volunteers must have no signs of infection and must not have taken antibiotics or other drugs that affect the immune system within 3 months before the study.

### 2.2. Sample Collection

The study material consisted of venous blood collected from the ulnar vein into a 4.9-mL tube containing the anticoagulant ethylenediaminetetraacetate tripotassium (EDTA-3K) and a 4.9-mL heparinized tube. A CBC with differential, including AMC (G/L) and ALC (G/L), was performed from an EDTA-3K tube using an automated hematology analyzer (Sysmex XN 1500). MLR was calculated by dividing the total number of monocytes by the total number of lymphocytes. The characteristic of CLL patients on MLR-high and MLR-low is presented in Table 1. Peripheral blood samples collected in heparinized tubes were used to detect leukemic cells and isolate peripheral blood mononuclear cells (PBMC), among which monocyte subpopulations were detected by flow cytometry. The material was analyzed within 2 h of collection.

Flow cytometry evaluation of leukemic B-lymphocytes (CD19+CD5+) with surface expression of CD38 and intracellular expression of ZAP-70 was performed from whole blood of CLL patients as previously described [25].

### 2.3. Isolation of PBMCs

Peripheral blood samples collected in heparinized tubes were used for density gradient centrifugation on Gradisol L (9003.1, Aqua-Med, Łódź, Poland) for 20 min at 700× g at room temperature. A layer of PBMCs was harvested as the interphase between Gradisol L and the diluted plasma. Isolated PBMCs were washed twice in PBS, and then counted in a Neubauer chamber.

### 2.4. Analysis of Monocyte Subsets by Flow Cytometry

Flow cytometry was used to differentiate monocyte subpopulations in PBMCs and detect cytokine expression ex vivo in monocyte subpopulations. Live PBMCs were distinguished from dead mononuclear cells using ViaKrome 808 Fixable Viability Dye (C36628, Beckman Coulter, CA, USA) according to the manufacturer's instructions. Viability below 95% disqualified PBMCs from further studies.

**Figure 1:**
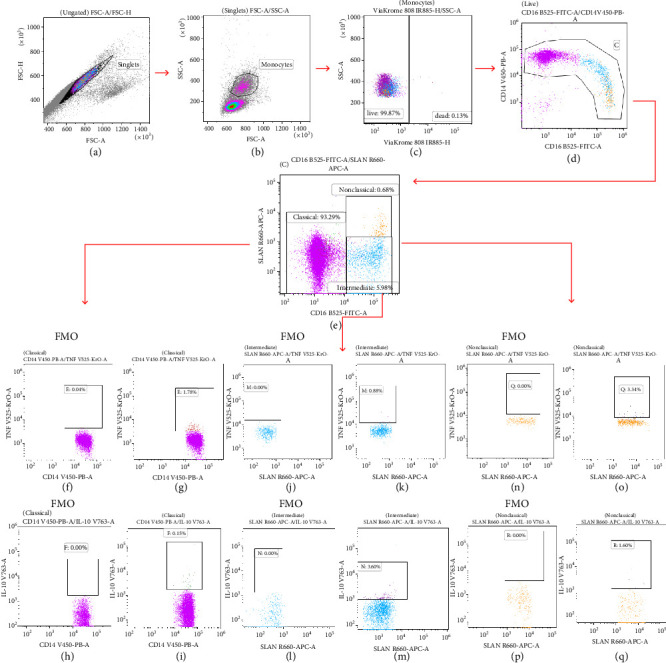
Example illustrating the gating during the cytometric analysis of monocyte subpopulations with intracellular expression of TNF and IL-10. Dot plot of FSC-H versus FSC-A—singlets separation (a). Dot plot SSC versus FSC gating of monocytes (b). Dot plot: ViaKrome 808 versus SSC—exclusion of dead cells from further analysis. Only live cells were used for further evaluation (c). Differentiation of monocyte subpopulations by CD14 and CD16 expression (dot plot: CD16 FITC vs. CD14 V450) (d). Dot plot SLAN APC versus CD16 FITC allowed the identification of classical (CD14++/CD16−/SLAN−), intermediate (CD14+/CD16+/SLAN−), and nonclassical (CD14+/CD16++/SLAN+) monocytes (e). Dot plots Fluorescence Minus One (FMO) are control tubes containing all panel antibodies minus one for TNF BV510 (f), (j), (n) or for IL-10 BV786 (h), (l), (p). Selected classical monocytes with intracellular expression of TNF BV510 (g) or IL-10 BV786 (i). Dot plot showing the percentage of intermediate monocytes TNF-positive (k) or IL-10-positive (m). Dot plot revealing percentage of nonclassical monocytes with intracellular expression of TNF (o) or IL-10 (q).

The 1 × 10^6^ live PBMCs were incubated with monoclonal antibodies: Mouse Anti-Human CD14 V450 (560349, MφP9, BD Biosciences) and Mouse Anti-Human CD16 FITC (555406, 3G8, BD Biosciences). Before the addition of the staining antibodies, anti-FcγR antibodies were used to prevent nonspecific binding to the Fc receptor (564219, BD Biosciences). The prepared cells were incubated for 10 min at room temperature, followed by incubation with mouse anti-human SLAN (M-DC8) APC (130-119-865, DD-1, Miltenyi Biotec, Bergisch Gladbach, NRW, Germany) for a further 10 min at 2°C–8°C. The cells were then washed twice with PBS (5 min; 700× g). After the labeling of monocyte surface markers, the cells were fixed (with Cytofix/Cytoperm)—10-min incubation and permeabilized (with Perm/Wash) (554714, BD Biosciences). Next, monocytes were incubated with monoclonal antibodies: Mouse Anti-Human TNF BV510 (502950, MAb11, BioLegend) or Rat Anti-Human IL-10 BV768 (564049, JES3-9D7, BD Biosciences) for 1 h (in the darkness, 2°C–8°C) and finally washed (5 min, 700× g) with PBS solution [26]. Samples were analyzed on a CytoFLEX LX flow cytometer (Beckman Coulter, CA, USA) with CytExpert software (Beckman Coulter, CA, USA). Kaluza Analysis software 2.1.1 (Beckman Coulter, CA, USA) was used to analyze and graph the collected data. The Fluorescence Minus One (FMO) control was used to set proper gates. The detailed gating strategies used for the flow cytometric analysis of intracellular TNF and IL-10 expression in classical, intermediate, and nonclassical monocyte subpopulations are shown in Figure 1.

### 2.5. FACS Sorting of Monocyte Subsets for RNA Isolation

FACS sorting of monocyte subpopulations was performed in 20 randomly selected CLL patients and 10 healthy volunteers. Monocyte subpopulations CD14++CD16−SLAN−, CD14+CD16+SLAN−, and CD14+CD16++SLAN+ after labeling with the anti-CD14 PE (345785, MφP9, BD Biosciences), anti-CD16 FITC (555406, 3G8, BD Biosciences), and anti-SLAN APC (M-DC8) (130-119-865, DD-1, Miltenyi Biotec) were sorted using BD FACSAria II (BD Biosciences). Monocyte subpopulations were collected at > 95% purity. The purity of each sorting was assessed shortly thereafter. RNA was isolated from classical, intermediate, and nonclassical monocytes sorted. Total RNA was isolated using the QIAamp RNA Blood Mini Kit (Cat 52304; Qiagen, Inc., Valencia, CA, USA) [26].

### 2.6. Analysis of MicroRNAs Expression by RT-qPCR

MicroRNA expression was analyzed using TaqMan MicroRNA Assays (Applied Biosystems, Foster City, CA, USA) and RT-qPCR according to the manufacturer's protocol. The TaqMan MicroRNA RT Kit (4366596, Thermo Fisher Scientific, Waltham, MA, USA) was used for reverse transcription. The reaction was performed on a total RNA from classical, intermediate, and nonclassical monocytes with miRNA-specific stem-loop primers. The resulting complementary DNA (cDNA) was used for microRNA expression analysis, carried out by using TaqMan Universal PCR Master Mix II (4440040, Thermo Fisher Scientific, Waltham, MA, USA) and specific TaqMan probes. The following set of three TaqMan MicroRNA Assays was used: hsa-miR-21-3p (002438, Thermo Fisher Scientific, Waltham, MA, USA), hsa-miR-150-5p (000473, Thermo Fisher Scientific, Waltham, MA, USA), hsa-miR-106a-5p (002169, Thermo Fisher Scientific, Waltham, MA, USA). RT-qPCRs were performed using the Applied Biosystems 7300 Real-Time PCR System (Thermo Fisher Scientific, Applied Biosystems, Inc., Waltham, MA, USA). The level of the analyzed miRNA expression was normalized to hsa-miR-16-5p (000391, Thermo Fisher Scientific, Waltham, MA, USA) as endogenous control and the relative expression of miRNAs was calculated using the 2^−∆∆Ct^ formula [27].

### 2.7. Statistical Analysis

Statistical analyses were conducted by utilizing GraphPad Prism software (Version 8.2.1, San Diego, CA, USA) and Statistica software (Version 13.3. StatSoft, Cracow, Poland). The D'Agostino & Pearson test was used to analyze the data distribution. The results are presented as: median, interquartile range (Q3-Q1) (IQR). The Mann–Whitney *U* test (for the comparison of two independent groups) or the Kruskal–Wallis test followed by Dunn's post hoc test (for the comparison of three independent groups) was used for data with other than normal distribution. Time-to-treatment (TTT) curves were obtained using the Kaplan–Meier method. The log-rank test was used to determine differences between groups. Receiver operating characteristics (ROC) analysis and the Youden index method were used to calculate the optimal cutoff value of the MLR that best discriminated between ZAP-70-positive and ZAP-70-negative CLL patients. The area under the curve (AUC) was also estimated. *p*-values < 0.05 were considered statistically significant.

## 3. Results

### 3.1. CLL Favors Decreased Value of MLR in Blood but Increases in CLL Patients With Advanced Clinical Stages

**Figure 2:**
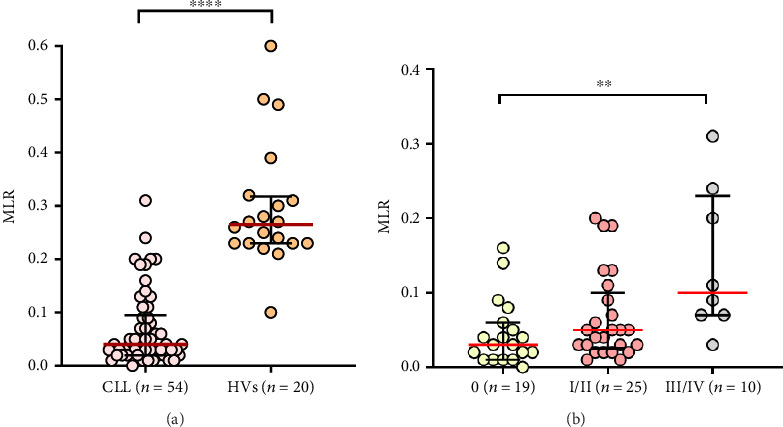
The comparison of the MLR values in CLL patients and HVs (a). Comparison of MLR values in CLL patients in different disease clinical Rai stages. (b) The median is marked with a solid red line. The whiskers represent the IQR. Variables were compared using the Mann–Whitney U test and the Kruskal–Wallis test with Dunn correction; ^∗∗∗∗^*p* < 0.0001, ^∗∗^*p* < 0.01, MLR, monocyte-to-lymphocyte ratio, HVs, healthy volunteers.

CLL patients had a significantly lower MLR (median, IQR; 0.04, 0.095–0.02) compared to healthy donors (median, IQR; 0.265, 0.318–0.23) (*p* < 0.0001) (Figure 2(a)). In the study group, we observed that the MLR was significantly higher in CLL patients with stage III/IV (median, IQR; 0.1, 0.23–0.07) compared to CLL patients classified to stage 0 (median, IQR; 0.03, 0.06–0.01) (*p* < 0.01) (Figure 2(b)).

### 3.2. The MLR Index Increases in CLL Patients With Worse Prognostic Factors

An increasing number of prognostic factors are being used to predict the course of CLL [28]. A poorer prognosis is associated with, among other parameters, a more advanced clinical stage of Rai, elevated β2-microglobulin levels and overexpression of CD38 and ZAP-70 in leukemic lymphocytes [29]. The ZAP-70 expression level in CLL cells is relatively stable over the course of the disease, whereas CD38 expression appears to be variable [30, 31]. Furthermore, the expression of these two markers has been shown to correlate with *IGHV* mutation status in CLL patients [30–32].

**Figure 3:**
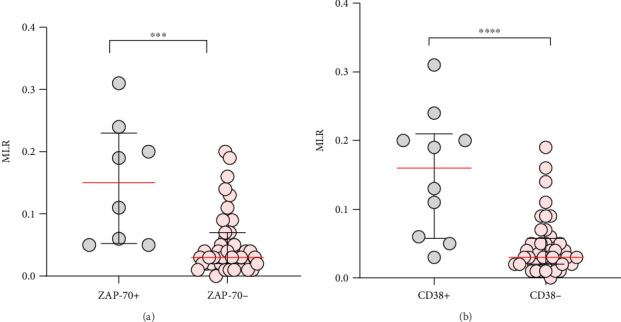
Distribution of MLR values in CLL patients differentiated based on ZAP-70 expression in the leukemic cells into ZAP-70− and ZAP-70+ groups (a). CD38 expression on leukemic cells into CD38+ and CD38− CLL patients (b). The median is marked with a solid red line. The whiskers represent the IQR. Variables were compared using the Mann–Whitney U test. A cutoff point for ZAP-70+ in CD19+/CD5+ cells was ≥ 20% and CD38+ in CD19+/CD5+ cells was ≥ 30%. ^∗∗∗^*p* < 0.001, ^∗∗∗∗^*p* < 0.0001.

In addition, a significant 5-fold increase in MLR was observed in the group of ZAP-70+ CLL patients (median, IQR; 0.15, 0.23–0.05) detected at diagnosis compared to ZAP-70− (median, IQR; 0.03; 0.07–0.02) (*p* < 0.001) (Figure 3(a)). A similar observation was made in CD38+ CLL patients (median, IQR; 0.16, 0.21–0.06) versus CD38− ones (median, IQR; 0.03, 0.058–0.02) (*p* < 0.0001) (Figure 3(b)).

To determine the optimal cutoff point of the MLR that best discriminates between ZAP-70+ and ZAP-70− CLL cases, ROC analysis was performed using Youden's index. ZAP-70 expression in the CLL cells is one of the negative prognostic factors for CLL and is a diagnostic feature for CLL patients with a more severe clinical course.

**Figure 4:**
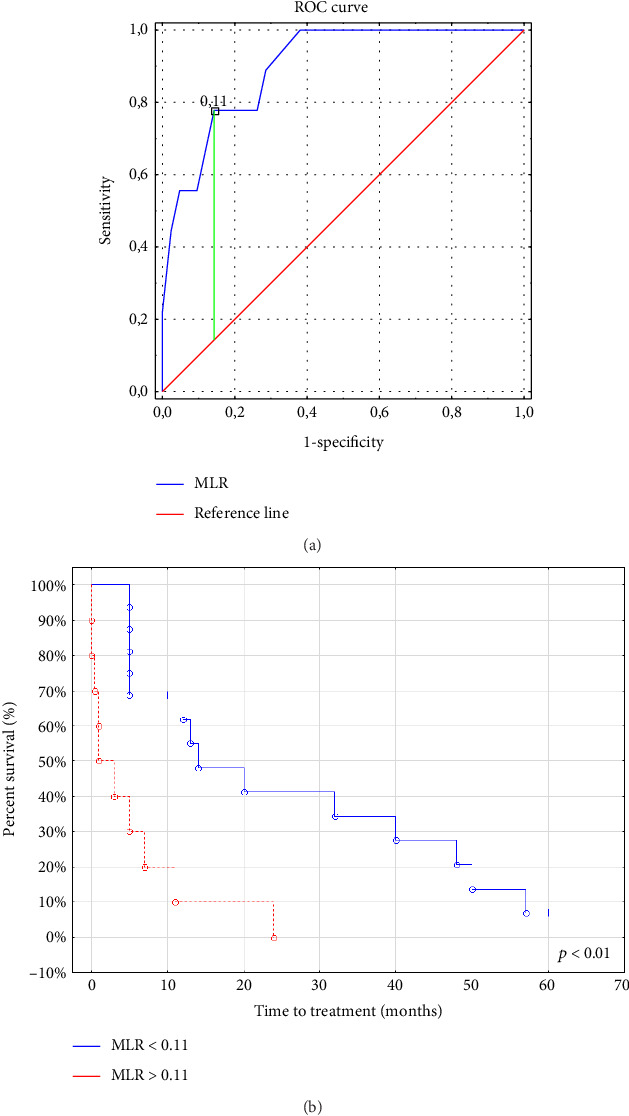
ROC curve analysis was useful for the determination of the cutoff point for MLR values differentiating ZAP-70-positive from ZAP-70-negative CLL patients. The red line is the ROC curve reference line. The blue line represents the performance of the MLR in distinguishing between ZAP-70-positive and ZAP-70-negative CLL patients. The green line marks the MLR threshold that best separates ZAP-70-positive from ZAP-70-negative CLL patients (a). Kaplan–Meier survival curves were used to assess the difference between the MLR-low and MLR-high CLL patients for time to first treatment (TTT) (b). Time-to-treatment initiation was statistically significant different between the MLR < 0.11 (MLR-low) and MLR > 0.11 (MLR-high) groups (*p*=0.007).

The AUC was also estimated. The optimal threshold for MLR was 0.11 (AUC, 0.899; SE 0.049, 0.804–0.995; *p*  <  0.0001) (Figure 4(a)).

### 3.3. Percentage Distribution of Monocyte Subpopulations in MLR-High and MLR-Low CLL Patients Considering the Division According to the Expression of ZAP-70 and CD38

To evaluate the coexisting association between MLR, a biomarker of inflammation in the blood, and poor prognosis in CLL patients, we investigated differences in percentage of subpopulations of classical, intermediate, and nonclassical monocytes in the peripheral blood of CLL patients using flow cytometry. The gating strategy is present in Figure 1.

**Figure 5:**
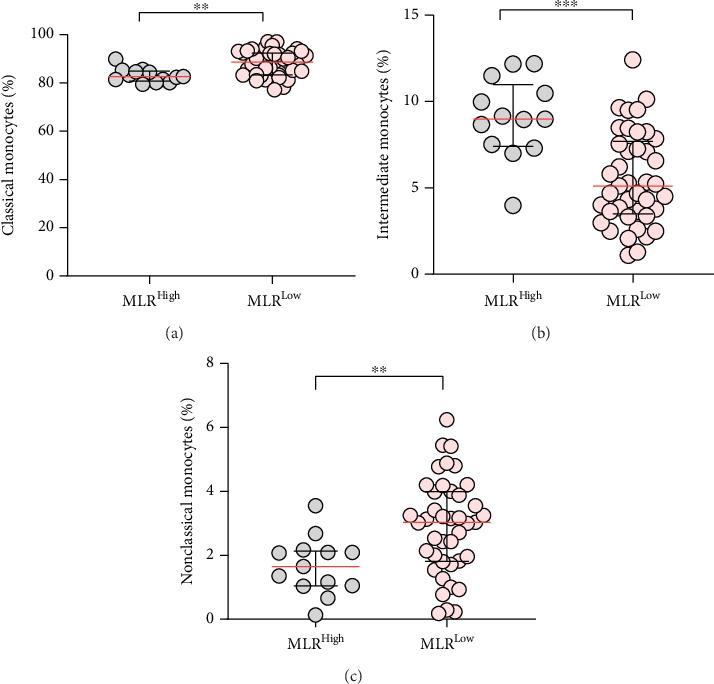
The differentiation of percentages of monocytes subpopulation of classical monocytes (a), intermediate monocytes (b), and nonclassical monocytes (c) in groups of MLR-high and MLR-low CLL patients. The median is marked with a solid red line. The whiskers represent the IQR. Variables were compared using the Mann–Whitney U test. ^∗∗^*p* < 0.01, ^∗∗∗^*p* < 0.001.

The study showed that CLL patients with the MLR-low index had a significantly higher percentage of classical and nonclassical monocytes compared to CLL patients with the MLR-high (median, IQR; 88.62, 92.37–83.36 and median, IQR; 3.03, 3.99–1.81 vs. median, IQR; 82.64, 84.92–80.78 and median, IQR; 1.65, 2.13–1.04; *p* < 0.01) (Figures 5(a), 5(c)).

In turn, the percentage of intermediate monocytes in the MLR-high group is significantly higher (median, IQR; 8.98, 10.99–7.405) than in the MLR-low CLL patients (median, IQR; 5.10, 7.69–3.50; *p* < 0.001). This suggests that the inflammation manifested in the blood of CLL may be an important determinant of monocyte phenotype differentiation (Figure 5(b)).

**Figure 6:**
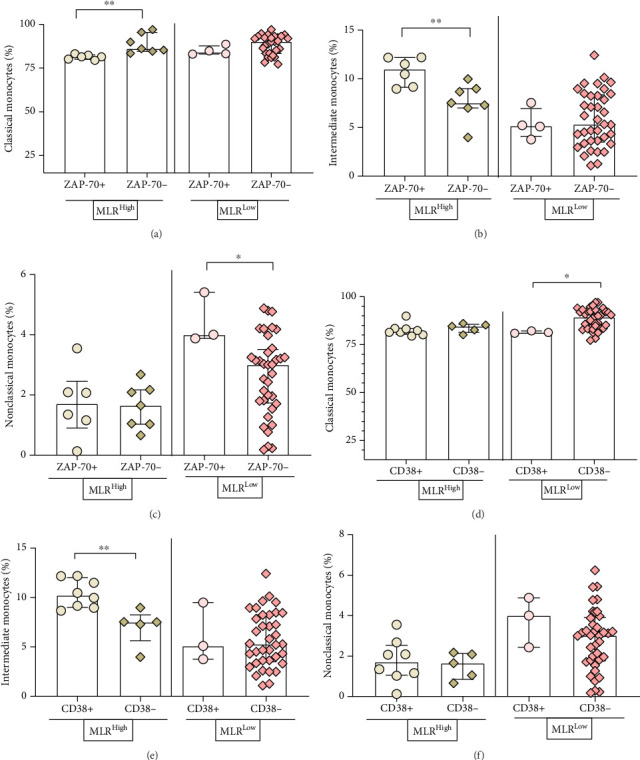
Comparison of the percentage of classical (a), (d), intermediate (b), (e), and nonclassical (c), (f) monocytes depending on ZAP-70 and CD38 expression on leukemic cells in MLR-high and MLR-low CLL patients. The median is represented by the top line of the bar. The whiskers represent the IQR. Variables were compared using the Mann–Whitney U test. A cutoff point for ZAP-70+ in CD19+/CD5+ cells was ≥ 20% and CD38+ in CD19+/CD5+ cells was ≥ 30%. ^∗^*p* < 0.05; ^∗∗^*p* < 0.01.

Moreover, the study showed a significantly higher percentage of classical monocytes in ZAP-70- compared to ZAP-70+ CLL patients with an elevated MLR index (median, IQR; 86.06, 95.46–84.58 vs. 81.44, 82.55–79.96; *p*=0.0012) (Figure 6(a)). However, the percentage of classical monocytes was significantly higher in CD38− versus CD38+ CLL patients with low MLR (median, IQR; 89.34, 92.94–83.47 vs. 81.34, 82.19–80.91; *p*=0.0147) (Figure 6(d)).

The percentage of intermediate monocytes in groups ZAP-70+ and CD38+ were significantly higher than ZAP-70− and CD38− in MLR-high CLL patients (median, IQR; 10.99, 12.18–9.11, vs. 7.51, 8.98–7.0, *p*=0.0082 and median, IQR; 10.23, 12.0–9,01 vs. 7.51, 8.26–5.64, *p*=0.0062) (Figures 6(b), 6(e)).

There was also a significantly higher percentage of nonclassical monocytes in ZAP-70+ compared to ZAP-70− CLL patients with MLR-low value (median, IQR; 4.0, 5.41–3.88 vs. 3.0, 3.51–1.73, *p*=0.0378 (Figure 6(c)).

### 3.4. Intracellular Expression of Selected Pro- and Anti-Inflammatory Cytokines in Classical, Intermediate, and Nonclassical Monocyte Subpopulations in MLR-High and MLR-Low CLL Patients

We then assessed the ability of monocyte subpopulations to produce selected cytokines.

**Figure 7:**
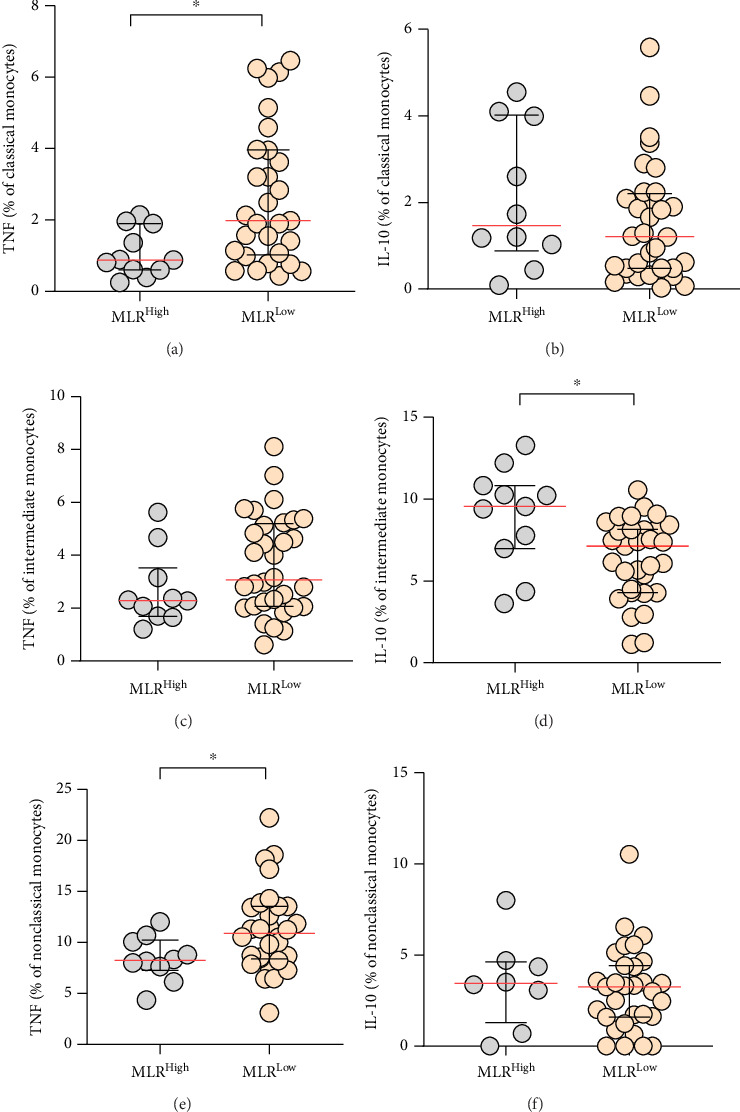
Percentage distribution of classical (a), (b), intermediate (c), (d), and nonclassical (e), (f) monocytes with intracellular expression of TNF and IL-10 in MLR-high and MLR-low CLL patients. The median is marked with a solid red line and the bar plot. The whiskers represent the IQR. Variables were compared using the Mann–Whitney U test. ^∗^*p* < 0.05.

In the MLR-low CLL patients, the percentage of classical monocytes with TNF expression (median, IQR; 1.98, 3.96–1.03) was significantly higher than in the MLR-high CLL group (median, IQR; 0.88, 1.9–0.6; *p*=0.0125) (Figure 7(a)). Moreover, the percentage of nonclassical monocytes with intracellular TNF expression was significantly higher in the MLR-low group compared to the MLR-high group (median, IQR; 10.89, 13.54–8.39 vs. 8.24, 10.23–7.26; *p*=0.043) (Figure 7(e)).

Furthermore, of all the monocyte subpopulations analyzed, only the percentage of intermediate monocytes with intracellular expression of IL-10 was significantly higher in the MLR-high group compared to the MLR-low CLL group (median, IQR; 9.55, 10.82–6.98 vs. 7.13, 8.15–4.29; *p*=0.0103) (Figure 7(d)).

**Figure 8:**
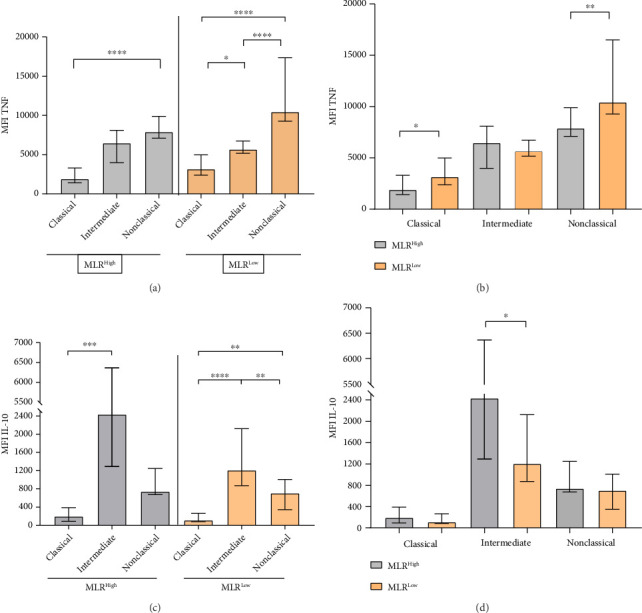
Intracellular expression of TNF (a), (b) and IL-10 (c), (d) presented by MFI in classical, intermediate, and nonclassical monocytes in MLR-high and MLR-low CLL patients. The median is marked with a solid red line and the bar plot. The whiskers represent the IQR. Variables were compared using the Kruskal–Wallis test followed by Dunn's post hoc test. ^∗^*p* < 0.05, ^∗∗^*p* < 0.01, ^∗∗∗^*p* < 0.001, ^∗∗∗∗^*p* < 0.0001.

In addition, we analyzed the levels of TNF and IL-10 expressions (MFI) in each monocyte subpopulation in MLR-high and MLR-low CLL patients. Among all monocyte subpopulations evaluated, nonclassical monocytes showed the significantly highest intracellular expression of TNF, but intermediate intracellular expression of IL-10 in MLR-low and MLR-high CLL patients (Figures 8(a), 8(c)).

We observed significantly higher expression of TNF presented by MFI in nonclassical monocytes in MLR-low CLL patients compared to nonclassical monocytes in MLR-high groups (median, IQR; 10434, 16502-9268 vs. 7886, 9882-7094; *p* = 0.005) (Figure 8(b)). On the other hand, the intracellular expression of IL-10 presented by MFI was significantly higher in intermediate monocytes from MLR-high CLL patients compared intermediate monocytes from MLR-low groups (median, IQR; 2433, 6365-1293 vs. 1207, 2123-870.3; *p* = 0.011) (Figure 8(d)).

We then assessed the percentage of classical and nonclassical monocytes with intracellular TNF expression and the percentage of intermediate monocytes with intracellular IL-10 expression between ZAP-70+, ZAP-70− and CD38+, CD38− CLL patients with low and high MLR index.

**Figure 9:**
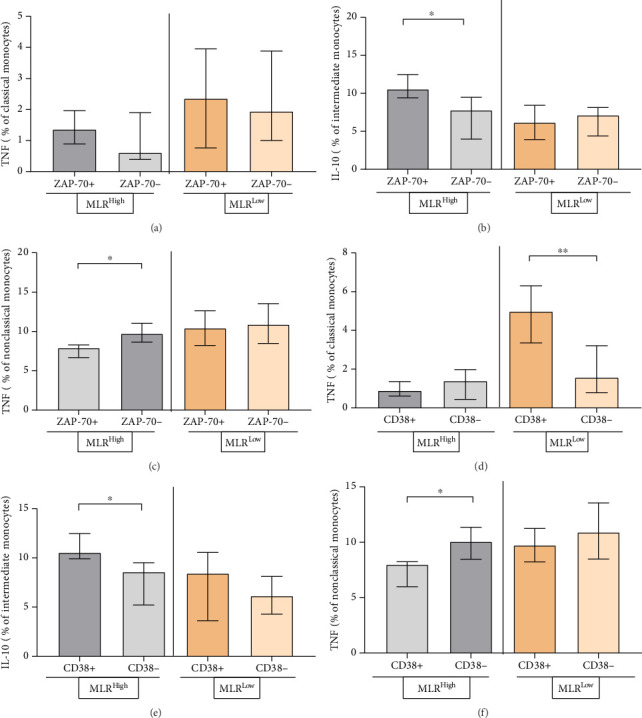
The differentiation of percentage of classical (a), (d) and nonclassical (c), (f) monocytes with intracellular expression of TNF and intermediate monocytes with intracellular IL-10 (b), (e) expression in ZAP-70+, ZAP-70−, CD38+, and CD38− in MLR-high and MLR-low CLL patients. The median is marked with the bar plot. The whiskers represent the IQR. The whiskers represent the IQR. Variables were compared using the Mann–Whitney U test. A cutoff point for ZAP-70+ in CD19+/CD5+ cells was ≥ 20% and CD38+ in CD19+/CD5+ cells was ≥ 30%. ^∗^*p* < 0.05, ^∗∗^*p* < 0.01.

The percentage of classical monocytes with intracellular TNF expression was significantly higher in CD38+ MLR-low CLL patients than in CD38− MLR-low CLL patients (median, IQR; 4.97, 6.29–3.35 vs. 1.57, 3.2–1.57; *p*=0.0051) (Figure 9(d)). However, the percentage of intermediate monocytes with intracellular IL-10 expression in MLR-high CLL patients was significantly higher in the ZAP-70+ group compared to the ZAP-70− group (median, IQR, 10.55, 12.47–9.41 vs. 7.78, 9.47–3.98; *p*=0.0303) (Figure 9(b)) and in the CD38+ group compared to the CD38− group (median, IQR, 8.42, 10.56–3.62 vs. 6.14, 8.12–4.28; *p*=0.0381) (Figure 9(e)). On the other hand, the percentage of nonclassical monocytes in MLR-high CLL patients was significantly higher in the ZAP-70- group compared to the ZAP-70+ group (median, IQR, 9.71, 11.04–8.64 vs. 7.89, 8.28–6.67; *p*=0.0381) (Figure 9(c)). The similar significant difference in the percentage of nonclassical monocytes was observed between groups CD38− and CD38+ (median, IQR, 10.07, 11.37–8.47 vs. 8.00, 8.24–5.99, *p*=0.0317) (Figure 9(f)).

### 3.5. miR-106a-5p, miR-150-5p, and miR-21-3p Expression in Monocyte Subpopulations of CLL Patients and Healthy Donors

**Figure 10:**
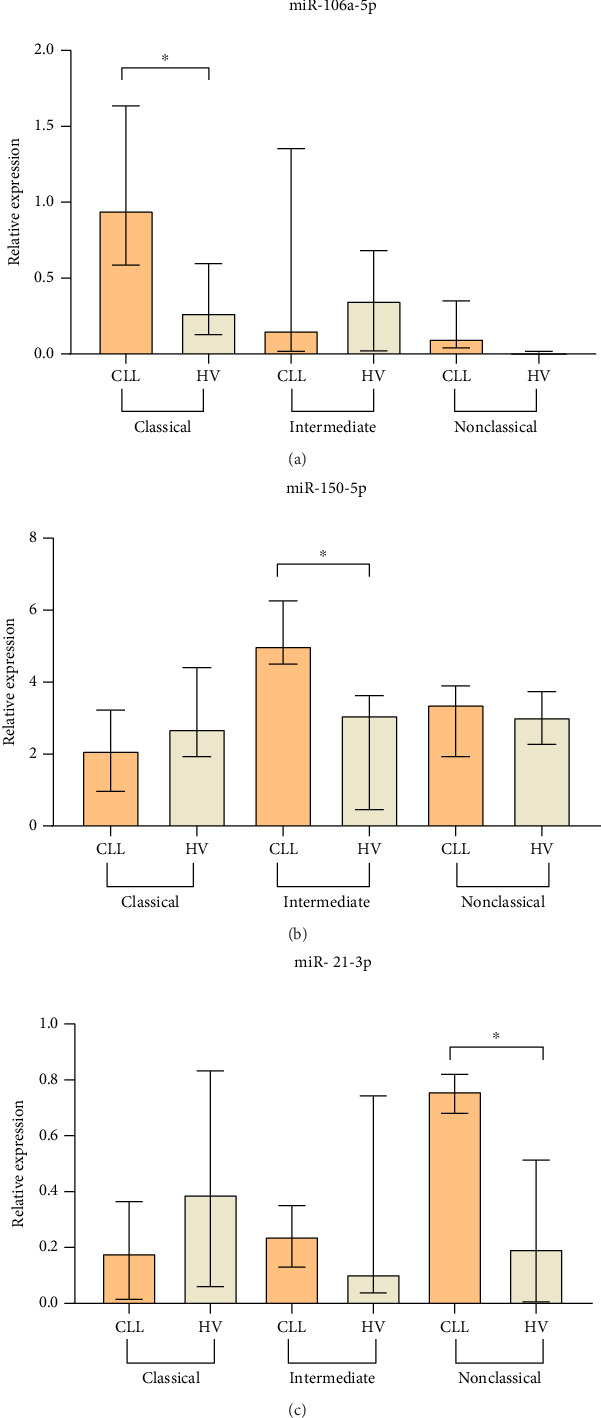
The relative expression of miRNA: miR-106a-5p (a), miR-150-5p (b), and miR-21-3p (c) in purified classical, intermediate, and nonclassical monocytes from CLL patients and healthy volunteers. The median is marked with the bar plot. The whiskers represent the IQR. Variables were compared using the Mann–Whitney U test. CLL, chronic lymphocytic leukemia, HV, healthy volunteers, ^∗^*p* < 0.05.

We investigated the expression levels of miR-21-3p, miR-150-5p, and miR-106a-5p in FACS-sorted classical, intermediate, and nonclassical monocytes in CLL patients and healthy volunteers. Our results show that significantly higher expression of miR-106a-5p was detected in classical monocytes from CLL patients compared to classical monocytes from healthy volunteers (median, IQR; 0.95, 1.65–0.585 vs. 0.27, 0.60–0.13; *p*=0.0293) (Figure 10(a)). Moreover, the expression of miR-106a-5p was higher in classical CLL-associated monocytes compared to other monocyte subpopulations (Figure 10(a)). The relative expression of miR-150-5p was significantly higher in intermediate monocytes from CLL patients compared to intermediate monocytes from healthy donors (median, IQR; 5.01, 6.26–4.5 vs. 3.08, 3.63–0.46; *p*=0.014) (Figure 10(b)). In turn, nonclassical monocytes from CLL patients are characterized by a significantly higher expression of miR-21-3p compared to healthy ones (median, IQR; 0.76, 0.82–0.68 vs. 0.2, 0.51–0.01; *p*=0.0242) (Figure 10(c)).

**Figure 11:**
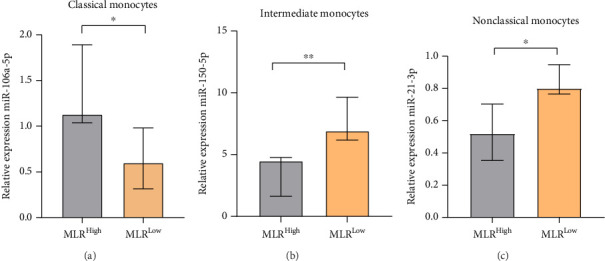
miR-106a-5p expression in classical monocytes (a), miR-150-5p expression in intermediate (b), and miR-21-3p expression in nonclassical monocytes (c) in MLR-high and MLR-low CLL patients. The median is marked with the bar plot. The whiskers represent the IQR. Variables were compared using the Mann–Whitney U test. ^∗^*p* < 0.05, ^∗∗^*p* < 0.01.

Based on the above results, we also decided to evaluate the difference in relative expression of miR-106a-5p in classical, miR-150-5p in intermediate, and miR-21-3p in nonclassical monocytes from MLR-high and MLR-low CLL patients. We observed a significantly higher miR-106a-5p expression in classical monocytes in MLR-high CLL patients compared to classical monocytes in MLR-low groups (median, IQR; 1.13, 1.89–1.04 vs. 0.6, 0.98–0.32, *p*=0.0317) (Figure 11(a)). On the other hand, the relative expression of miR-150-5p in intermediate monocytes and miR-21-3p in nonclassical monocytes was significantly lower in MLR-high CLL patients compared to MLR-low ones. Moreover, we found that the level of miR-150-5p in intermediate monocytes was the highest in MLR-low CLL patients compared to MLR-high CLL patients (median, IQR; 6.92, 9.64–6.18 vs. median, IQR; 4.5, 4.77–1.63, *p*=0.0079) (Figure 11(b)). In addition, nonclassical monocytes from MLR-low CLL patients are characterized by a significantly higher expression of miR-21-3p compared to nonclassical monocytes from MLR-high CLL groups (median, IQR; 0.8, 0.94–0.76 vs. 0.52, 0.7–0.35, *p*=0.0310) (Figure 11(c)).

### 3.6. miR-106a-5p, miR-150-5p, and miR-21-3p Expression in Classical, Intermediate, and Nonclassical Monocytes From CLL Patients in Relation to the Expression of ZAP-70 and CD38

**Figure 12:**
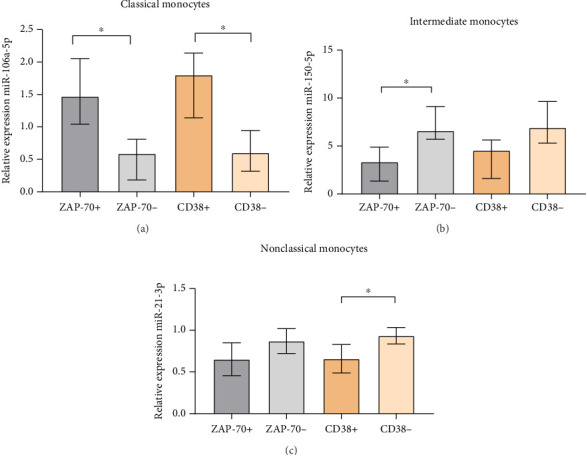
miR-106a-5p expression in classical monocytes (a), miR-150-5p expression in intermediate (b), and miR-21-3p expression in nonclassical monocytes (c) in ZAP-70-positive, ZAP-70-negative, CD38-positive, and CD38-negative CLL patients. The median is marked with the bar plot. The whiskers represent the IQR. Variables were compared using the Mann−Whitney U test. A cutoff point for ZAP-70 + in CD19+/CD5+ cells was ≥ 20% and CD38+ in CD19+/CD5+ cells was ≥ 30%. ^∗^*p* < 0.05.

Moreover, we observed a significantly higher expression of miR-106a-5p in classical monocytes from ZAP-70+ compared to ZAP-70− CLL patients (median, IQR; 1.47, 2.05–1.04 vs. 0.59, 0.81–0.18; *p*=0.0286) and in CD38+ CLL patients compared to CD38− CLL patients (median, IQR; 1.8, 2.14–1.14 vs. 0.6, 0.95–0.315; *p*=0.0357) (Figure 12(a)).

Among CLL-associated intermediate monocytes, a significantly higher expression of miR-150a-5p was observed in ZAP-70− patients compared to ZAP-70+ CLL patients (median, IQR; 6.59, 9.12–5.7 vs. 3.35, 4.89–1.35, *p*=0.0381) (Figure 12(b)). In addition, a significantly higher expression of miR-21-3p from CLL nonclassical monocytes was in the CD38− group compared to CD38+ (median, IQR; 0.99, 0.99–0.82 vs. 0.71, 0.77–0.52, *p*=0,0357) (Figure 12(c)).

## 4. Discussion

It is crucial to have a thorough understanding of the influence of three main factors: inflammation, the microenvironment, and biology of leukemic cells to understand the mechanisms of leukemia initiation and progression [1]. Monocytes appear to be an important component of the cellular response in CLL [33]. They are one of the key regulators of inflammation and are one of the most variable groups of cells in the TME [34]. However, in CLL, an elevated absolute number of monocytes at the time of diagnosis is an unfavorable prognostic factor [35, 36]. The processes underlying the effects of inflammation on hematological malignancies are still not fully understood [2]. Blanco et al. [37] have observed a decreased inflammatory response, mainly involving monocytes, in the early stage of CLL patients compared to monoclonal B lymphocytosis (MBL) patients [37]. In the study by Blanco et al. [37], monocyte subpopulations were not isolated to characterize their pro- or anti-inflammatory properties in MBL and early CLL, which in our opinion was a significant shortcoming. Therefore, it is noteworthy that our study focused on the separate evaluation of the three main monocyte subpopulations in CLL patients. Unfortunately, the molecular and functional assessment of monocyte subpopulations in CLL is difficult in daily clinical practice. Therefore, the discovery of inexpensive, simple, and reliable hematological parameters that reflect the activity of monocyte subpopulations in CLL patients would be extremely helpful in assessing disease stability [38, 39]. Lately, Yokus et al. [40] and Chiarenza et al. [41] demonstrated the association between increased LMR, NLR, and shorter TTT CLL [40, 41]. This is a particularly important finding because it is the functional capacity of inflammatory cells relative to leukemic cells that can largely determine the disparity between the efficiency of pro- and antitumor response activity of microenvironment [42]. MLR is considered an early indicator of inflammation, the predictive value of which has been established in several lymphoproliferative diseases [43]. Shi et al. [44] showed that MLR ≥ 0.3, the cutoff value in multiple myeloma patients, was associated with shorter progression-free survival [44]. Our study showed that an MLR ≥ 0.11 is associated with a higher risk of CLL progression and a shorter time to first treatment. This finding suggests that MLR is an inexpensive and easy-to-measure blood parameter that may help estimate the prospect of CLL burden. Circulating monocytes are important mediators of the inflammatory response and are the major source of cytokines. Le Gallou et al. [45] analyzed the surface markers and the gene profile in monocyte subsets suggesting that classical and intermediate monocytes represent the inflammatory phenotype educated by DLBCL [45]. In our study, we observed that CLL patients classified in the MLR-low group had a significantly higher percentage of TNF-positive classical and TNF-positive nonclassical monocytes compared to the MLR-high CLL patients. This supports the notion that they have an enhanced pro-inflammatory function in the microenvironment of indolent CLL. In contrast, the percentage of monocytes with an intracellular expression of IL-10 was significantly higher among intermediate monocytes in CLL patients with high-MLR values, suggesting their protumor properties promote CLL burden. Anti-inflammatory intermediate monocyte properties in CLL are contrary to the reports of the research group Le Gallou et al. [45] in DLBCL patients. This may be related to different inflammatory pathways; we focused on assessing the intracellular expression of pro- versus anti-inflammatory cytokines in circulating monocyte subsets, whereas Le Gallou assessed chemokine-dependent pathways [45]. These reports emphasize the importance of continuing research necessary to understand the paradoxical functions of each monocyte subpopulation in patients with hematological malignancies, particularly in patients with negative prognostic factors. In our study, in MLR-high CLL patients with poor prognostic factors, that is, increased expression of CD38 and ZAP-70 in leukemic cells, we observed a significantly lower percentage of nonclassical monocytes with intracellular TNF expression and a significantly higher percentage of intermediate monocytes with intracellular IL-10 expression, allowing us to see a correlation between the functional maturation of the circulating monocytes and the degree of disease progression. One of these may be an epigenetic look at monocyte subsets, with a particular focus on the role of miRNAs. In CLL, miRNAs have been identified as potent regulators that influence several cellular pathways in CLL, such as immunomodulation, cell proliferation, and cell death [46]. To date, they have mainly been identified in leukemic lymphocytes or CLL patient serum [47–49]. However, we sought to check three microRNAs expressed in FACS-sorted classical, intermediate, and nonclassical monocytes in CLL patients and healthy volunteers. miR-106a-5p levels were increased in serum patients with autoimmune diseases, including psoriasis [50], which was associated with promoting inflammation and the progression of the disease [50]. miR-106a-5p, a member of the miR-17 family, plays an important role in the downregulation of the TGF-β signaling pathway in CLL patients [51]. However, the exact role of miR-106a-5p in the phenotypic and functional profiling of monocyte subpopulations in CLL patients remains to be elucidated. Our studies have shown that MLR-high CLL patients have a reduced percentage of classical monocytes with intracellular TNF expression with significantly elevated miR-106a-5p expression, suggesting that miR-106a-5p may affect the dysfunction of classical monocytes responsible for monocytosis by regulating the ability of these cells to secrete TNF. Moreover, our studies appear to extend the existing importance of miR-106a-5p for monocytic lineage cell function developed by Zhu et al. [52]. The authors have previously shown that elevated levels of miR-106a-5p modulate the inflammatory properties of macrophages through a SIRP-dependent pathway, reducing their capacity for activation, phagocytosis, and secretion of proinflammatory cytokines [52]. Notably, our study found that CLL patients with worse prognostic factors, that is, high expression of ZAP-70 and CD38, had classical monocytes with high expression of miR-106a-5p, suggesting that this oncomiR may be one of the key regulators influencing the attenuation of antitumor activity of these cells, thus promoting disease progression. The results obtained may represent an alternative point of view to that presented by Mizahura et al. [53] The authors showed that miR-106a-5p, present in exosomes derived from myeloma cells after transfer to PBMC cells, including monocytes, plays an important role in modifying the expression of genes responsible for the development of inflammation and the formation of a subpopulation of monocytic cells with immunosuppressive properties, the so-called M-MDSC [53]. Shu (2018) showed that the expression of miR-150-5p in the THP-1 line monocytes by inhibiting Notch3 caused a decrease in the expression of the CCR2 on tumor-associated monocyte cells [54]. Cassabone et al. [55] demonstrated the prognostic role of serum circulating miR-150-5p in CLL patients [55]. However, the effect of miR-150-5p on monocytes from CLL is still unclear. Accordingly, we decided to assess levels of miR-150-5p among CLL-associated monocyte subsets. It is worth highlighting that Selimoglu-Buet [56] showed that in patients with chronic myelomonocytic leukemia (CMML), hsa-miR-150 expression in circulating monocytes was the highest in nonclassical monocyte subpopulations [56]. However, in our study, intermediate monocytes had the highest level of miR-150-5p expression, demonstrating the heterogeneous role of this molecule in cancer-associated monocyte subpopulations. In addition, we observed reduced miR-150-5p expression in the intermediate monocytes of ZAP-70+ and CD38+ patients, which may suggest an effect of miR-150-5p on the formation of intermediate monocytes to promote CLL. Connaughton et al. [57] showed that the intermediate monocyte phenotype is also characterized by reduced CCR2 receptor expression, making it conceivable that intermediate monocytes in CLL patients with low CCR2-dependent increased miR-150-5p expression, but such a hypothesis requires further extensive studies. Recently, miR-21 has become one of the most studied oncogenic microRNAs in the pathogenesis of solid and hematological cancers [58–61]. miR-21-3p also plays an important role in regulating macrophage functions [62]. However, the effect of this microRNA on monocyte polarization in CLL has not been elucidated. Ruiz-Lafuente et al. [63] observed increased expression of miR-21 (both 5p and 3p variants) in CLL cells exposed to IL-4 in vitro [63]. Our studies show that miR-21-3p had higher expression in nonclassical monocytes in CLL patients. Chen et al. [64] showed that miR-21-3p affects the pathways that regulate proinflammatory cytokine release, including SOCS4/STAT3 [64]. They found that miR-21-3p in macrophages binds to SOCS4 and thereby inhibits STAT3 phosphorylation, which is necessary for the polarization of macrophages to the M2 subtype. It is suggested that this is one of the factors influencing the maintenance of the pro-inflammatory profile of M1 [64].

The present study apart from its strengths suffers as well from several limitations. We are aware that the number of CLL patients was not very large, but given the prevailing pandemic, we were very selective in classifying the study group to exclude as far as possible any confounding factors. Furthermore, in designing the above study, we focused only on newly diagnosed CLL patients, so that by assessing the immune status of the different monocyte subpopulations at the time of CLL diagnosis in the context of inflammation assessed by MLR, we could exclude the influence of disease progression or treatment used. However, it is worth adding that our study suggests a previously undescribed potential association between phenotypic, functional, and miRNA-dependent differences in the monocyte subpopulation in MLR-low and MLR-high CLL patients. Furthermore, we have outlined a likely inflammation-dependent mechanism related to the plasticity of monocyte subpopulation function in the CLL blood microenvironment.

## 5. Conclusion

Based on the results obtained, it can be concluded that the hematological marker of inflammation—MLR—may be useful in obtaining information about the phenotypic and functional immune status of monocyte subpopulations circulating in the blood of CLL patients. MLR-high CLL patients showed a decrease in the percentage of pro-inflammatory nonclassical monocytes (SLAN+) and an increase in the proportion of intermediate monocytes (SLAN−) with potential anti-inflammatory capacity. Furthermore, we revealed that in CLL patients and healthy subjects in each of the monocyte subpopulations studied, the expression of miR-106a-5p, miR-150-5p, and miR-21-3p was significantly different, which may suggest that the inflammatory conditions prevailing in the course of CLL also affect the mechanisms dependent on these miRNAs determining monocyte subpopulation heterogeneity.

## Figures and Tables

**Table 1:** Characteristics of MLR-high and MLR-low CLL patients.

Characteristic	MLR-high *n* = 13	MLR-low *n* = 41
*Gender of CLL patients*		
Female, *n* (%)	2 (15%)	17 (41%)
Male, *n* (%)	11 (85%)	24 (59%)

*Stage of CLL according to Rai*		
0, *n* (%)	3 (23%)	16 (39%)
I/II, *n* (%)	6 (46%)	19 (46%)
III/IV, *n* (%)	4 (31%)	6 (15%)

ZAP-70+ patients^$^, *n* (%)	6 (46%)	4 (10%)
CD38+ patients^$^, *n* (%)	8 (62%)	3 (7%)

WBC count (G/L), median (IQR)	20.78 (16.61–26.08)	28.77 (18.99–52.14)
ALC (G/L), median (IQR)	17.67 (12.66–19.92)	20.69 (12.21–45.38)
AMC (G/L), median (IQR)	4.19 (2.45–4.85)^#^	0.635 (0.47–1.35)^#^
β_2_M (mg/dL), median (IQR)	4.02 (2.87–5.09)^∗^	2.19 (1.82–2.87)^∗^
LDH (IU/L), median (IQR)	259 (198–388)	202 (170–311)

*Note: n*—number group.

Abbreviations: β_2_M, β_2_-microglobulin; ALC, absolute lymphocyte count; AMC, absolute monocyte count; IQR, interquartile range; LDH, lactate dehydrogenase; WBC, white blood cells.

^$^The expression of ZAP-70 and CD38 molecules was assessed on CD19+/CD5+ cells. The cutoff values were 20% and 30%, respectively.

^∗^ *p* < 0.001.

^*#*^ *p*  < 0.0001.

## Data Availability

The data that support the findings of this study are available on request from the corresponding author. The data are not publicly available due to privacy or ethical restrictions.
